# Interparental Violence: Similarities and Discrepancies Between Narratives of Mothers and Their Children

**DOI:** 10.1007/s10826-015-0137-3

**Published:** 2015-02-01

**Authors:** Floor Barbera van Rooij, Winneke Aalken van der Schuur, Majone Steketee, Jodi Mak, Trees Pels

**Affiliations:** 1 University of Amsterdam, Nieuwe Prinsengracht 130, 1018 VZ Amsterdam, The Netherlands; 2 Verwey-Jonker Instituut, Utrecht, The Netherlands; 3 Free University, Amsterdam, The Netherlands

**Keywords:** Interparental violence, Discrepancies between informants, Mother-child reports, Parental functioning, Child functioning

## Abstract

Previous studies and intervention programs on interparental violence have relied largely on reports either solely from parents or solely from children. Nevertheless, the literature and the theoretical background provide indications of the existence of discrepancies between the narratives of parents and those of children. This study therefore focuses on similarities and differences between the narratives of mothers and those of their children with regard to the children’s exposure to interparental violence and its impact on child and parental functioning. In depth open interviews were conducted to assess the narratives of 36 mothers (27–59 years of age) and 43 of their children (17 boys and 26 girls; 9–25 years of age) who had experienced interparental violence in their past. A hierarchical coding system was used to code the interviews. Thereafter, the differences between mother and child narratives were analyzed based on the coded fragments. Few differences were found between the narratives with regard to parental functioning. We did find discrepancies, however, with regard to the children’s exposure to interparental violence and its impact on child functioning. Exploratory analyses showed relationships between the discrepancies and the severity of the violence and age of the children. More attention to these differences is essential in order to enhance our knowledge concerning the complex impact of violence on family members and to improve support geared to their specific needs.

## Introduction

Prevalence estimates indicate that in the Netherlands (the country in which this study was conducted), approximately 10 % of the population has been exposed to domestic violence. The most common type of domestic violence is interparental violence, which refers to violence occurring between parents (Van der Veen and Bogaerts 2010). Exposure to interparental violence has a major impact on the functioning of children and families (e.g., Holt et al. 2008). Children exposed to interparental violence are more vulnerable to adjustment problems. For example, these children show more internalizing, externalizing, and academic problems. Furthermore, exposure to interparental violence is associated with higher levels of emotional, physical, and sexual abuse of children (DeBoard-Lucas and Grych 2011; Holt et al. 2008; Kitzmann et al. 2003; Wolfe et al. 2003). With regard to parental functioning, mothers continuously struggle to control their parenting behaviors during periods of interparental violence (Pels et al. 2010; Peled and Gil 2011). To illustrate, it was found that mothers have trouble protecting their children. Reports show that they tried hard to focus on their parenting, restrain their partners, or compensate for their violence, in addition to protecting their children from exposure to the violence. Nevertheless, they often failed to prevent their children from being exposed to the interparental violence (Peled and Gil 2011). In addition, interparental violence has been associated with the emotional unavailability of mothers (Pels et al. 2010; Sturge-Apple et al. 2012). Moreover, several studies have indicated that the relationship between interparental violence and child functioning is moderated through, or even mediated by parental functioning (e.g., DeBoard-Lucas et al. 2010; Skopp et al. 2007; Sturge-Apple et al. 2012). For example, supportive parenting decreases children’s self-blame and that self-blame is apparent only when parents report high levels of coercive behavior (DeBoard-Lucas et al. 2010). Positive family functioning is thus an important protective factor in the development of adjustment problems in children.

It is important to note that different informants within the family may provide different accounts of the same incidents. In examining the complex dynamics within families, it is preferable to take note of the perspectives of multiple informants in order to get a more complete understanding of these dynamics (Cottrell et al. 2003; Pelegrina et al. 2003). Previous studies about interparental violence, however, have relied largely on reports either solely from parents or solely from children. Less is known about discrepancies between the narratives of parents and children with regard to exposure to interparental violence and its impact on child and family functioning. We found only two studies on domestic violence that included some comparison between reports of mothers and children (Levendosky and Graham-Bermann 2001; McCloskey et al. 1995). Levendosky and Graham-Bermann (2001) found low correlations between mother and child reports with regard to the children’s adjustment in the context of interparental violence. Nevertheless, they did not report on the specific differences between these informants. McCloskey et al. (1995) observed a low (but significant) overall correspondence in the report’s by mothers and children. They found moderate to large correlations between mothers and children in their reports about aggression within the family (father to mother, father to child, mother to child). Differences were found regarding parental support: while in the reports of mothers parental support wasn’t related to the extent of the family violence, the children in families with more family violence perceived less parental support.

Several studies in other fields of child and family functioning did reveal discrepancies between the reports of parents and those of their children (e.g., Ohannessian et al. 2000; Pelegrina et al. 2003; Shapiro 2004; Stice and Barrera 1995). To illustrate, correlations between the perceptions of parents and children are usually moderate or low with regard to the externalizing problems of children. Children report higher levels of externalizing problems compared to their parents (Stice and Barrera 1995). In addition, in the study by Ohannessian and colleagues children assessed the functioning of their families more negatively than their parents do. Children also report lower levels of family cohesion and family adjustment compared to their parents (Ohannessian et al. 2000).

Scholars have proposed several hypotheses in order to explain differences between the perceptions of parents and those of their children. The Intergenerational Stake Hypothesis (Bengtson and Kuypers 1971; Giarrusso et al. 1995) aims to explain variation in the perspectives of parents and children based on differences between generations (i.e., the “generation gap”). The assumption is that discrepancies between generations arise due to variations in motivations and levels of investment at different developmental periods. According to this hypothesis, older generations value generativity, the desire to engage in worthwhile and lasting efforts, like procreation and the guidance of the next generation (Erikson 1968). In contrast, younger generations eventually attempt to achieve independence. For example, parents’ need for generativity may lead them to maintain continuity between generations by investing in their children, while children’s desire for independence may lead them to invest less in their parents. Parents could therefore view their parenting behaviors or their relationships with their children more positively than their children do. The Social Structural Model (Risman and Park 1988) may provide additional explanation for differences between the narratives of parents and those of their children (Shapiro 2004). This model refers to the contribution of the social structural context, suggesting that parents and children have different structural positions in society. Discrepancies in perceptions and narratives could arise due to variations in opportunities and expectations based on their social structural positions. For example, children who perceive that society offers them sufficient opportunities are likely to feel more independent and report lower levels of investment by their parents. Another model focusing on differences in perspectives is the Attribution Bias Context Model (De Los Reyes 2011; De Los Reyes and Kazdin 2005). This model was originally developed in order to explain informant discrepancies in the clinical assessment of children, based on the actor-observer phenomenon (Jones and Nisbett 1972). Although this model attends to informant discrepancies in clinical assessment of children, it may also explain discrepancies between the narratives of parents and those of children exposed to interparental violence. Similar to the original model, children are actors and parents observers in narratives about child functioning. In this context, however, parents are actors and children observers in narratives about interparental violence and parenting behaviors. The model assumes that differences between actors and observers arise due to discrepancies in attributions and perspectives. According to this model, actors tend to attribute the causes of their problems to the context in which the behavior is exhibited, while discounting or disregarding their own disposition. The perspectives and reports of actors are therefore likely to focus more on the context. In contrast, observers tend to attribute the causes of the actors’ problems to the actors’ dispositions, while discounting or disregarding the context in which the behavior is exhibited. The perspectives and reports of observers are therefore likely to focus more on the negative behaviors of actors (De Los Reyes 2011; De Los Reyes and Kazdin 2005).

In sum, the Intergenerational Stake Hypothesis (Bengtson and Kuypers 1971; Giarrusso et al. 1995), the Social Structural Model (Risman and Park 1988; Shapiro 2004), the Attribution Bias Context Model (De Los Reyes 2011; De Los Reyes and Kazdin 2005), and the previous mentioned literature indicate that discrepancies are to be expected between the narratives of mothers and those of children exposed to interparental violence. Therefore, it is important that we investigate these differences to increase our understanding of the family dynamics in families exposed to interparental violence. Moreover, better understanding of possible discrepancies may provide guidelines for better policies. Until now, in most countries, children are not (yet) involved in the assessment and treatment procedures: professionals usually only speak with the parent(s) about the children (Mak and Steketee 2013). Reliance on a single informant could provide incomplete information about the exposure of children to interparental violence and its impact on the functioning of children and families. This is particularly concerning since policies based on such incomplete information may result in insufficient support for both parents and children (e.g., Cottrell et al. 2003; Levendosky and Graham-Bermann 2001; Hill and Jones 1997; Pelegrina et al. 2003). For example, underreporting by parents with regard to their children’s exposure to interparental violence may result in under-reacting to such exposure, as well as in ineffective support (Hill and Jones 1997). A higher awareness of the discrepancies between mothers’ and children’s perspectives may also be important for preventive reasons. When professionals and informal workers are trained to better communicate about interparental violence and its consequences with both mothers and children, a risk assessment might be made sooner to ensure the safety of the child (Mak and Steketee 2013).

The aim of the present study is to compare narratives of mothers and their children who have experienced interparental violence, with regard to the children’s exposure to interparental violence and its impact on child and parent functioning. We expect that their narratives would differ, based on the Intergenerational Stake Hypothesis, the Social Structural Model, and the Attribution Bias Context Model. For example, with regard to the Intergenerational Stake Hypothesis mothers may report more positively about their parenting than their children do, given their need for generativity, perceived expectations, and the attribution of their parenting problems to the context. In addition, according to the Social Structural Model children may report more positively about their own functioning than their mothers do, given their motivations, perceived expectations of society, and the attribution of their problems to the context and not to their own disposition. We also explore the association between the severity of the violence and the age of the child, and discrepancies between the narratives of mothers and children. As McCloskey et al. (1995) observed, the degree of violence might influence the accounts of mothers and children differently. Also, the cognitive and social-emotional capacities associated with the age of children, might influence their appraisal and evaluation of the violence, as well as their recollection and disclosure of violence (see Fosco et al. 2007; Hungerford et al. 2012; Jouriles et al. 2000; Thompson et al. 2007).

## Method

### Participants

This study is part of a broader research project conducted in the Netherlands, focusing on the influence of interparental violence on the child and on parental functioning (qualitative interview study and literature review). The current study involved secondary analyses on the qualitative interview transcripts of mothers and children of the same family (see Pels et al. 2010).

We adopted several inclusion criteria when recruiting participants. First, mothers must have experienced interparental violence in the past, but the violence should have ceased by the time of the study. Second, their children must have witnessed the violence and lived with their parents during the period of interparental violence. In the current study, children’s exposure to interparental violence refers to children’s awareness of interparental violence in the past. Children were categorized as witnesses if they had seen or heard interparental violence taking place, as well as if they had been exposed to the violence indirectly, as with being told about it by their mother or seeing her injuries (Kitzmann et al. 2003). Third, in order to improve the reliability of the memories, the last time that the interparental violence occurred must have been between 6 months and 3 years before the time of the study. Finally, the participants were required to indicate that they understood the purpose of the study and that they were willing and able to participate. Addiction, mental retardation, and severe mental problems were therefore criteria for exclusion.

In order to recruit mothers for the study, key individuals (e.g., professionals working in the field of IPV, parenting, youth or community services, and professionals or laypeople working with or having a wide network within the targeted communities), leaflets, and various media channels were used. Additionally, participants were asked whether they knew any other women who would be willing to cooperate. Also, several professional organizations collaborated to recruit mothers (e.g., *Steunpunt Huiselijk Geweld*—a domestic-violence service center—women’s shelters, and youth institutions). Children were recruited by their mothers.

The sample consisted of 36 mothers and 43 of their children (17 boys and 26 girls). Mothers ranged in age from 27 to 59 years (*M* = 41.11, *SD* = 7.03). In most cases, one child of each mother participated. In five cases, however, two children of the same mother were willing to participate, and one case involved three children of the same mother. The ages of the children ranged from 9 to 25 years (*M* = 15.35, *SD* = 4.02). The mother-child pairs in the sample differed with respect to the ethnic background of the mothers: 7 were native Dutch, the others were immigrants (N = 29; mostly from the Caribean area (Surinam, the Antillean islands), Turkey and Morocco; based on the mother’s country of birth, as well as that of her parent(s)). Of the participating mothers, 47 % (*n* = 17) had a low level of education; 39 % (*n* = 14) had an intermediate level of education, and 14 % (*n* = 5) had a high level of education. Of the children, 91 % (*n* = 39) were still in school, 7 % (*n* = 3) had jobs, and one child was at home, due to difficulties experienced at school. For 26 % of the children (*n* = 11), the highest level of education completed was elementary school, while 47 % (*n* = 20) had completed high school, 14 % (*n* = 6) had completed vocational education, and 12 % (*n* = 5) had completed higher education. Descriptions regarding the type, frequency, and duration of the mothers’ exposure to partner violence as reported by the mothers are provided in Table 1.

**Table 1:** Overview of the mothers’ descriptions of violence (*n* = 36), concerning the type, frequency, and duration of interparental violence

Type	N (%)	Frequency	N (%)	Duration	N (%)
Physical	30 (83)	Daily/Weekly	25 (69)	>5 years	23 (64)
Psychological	36 (100)	Monthly	7 (19)	1–5 years	12 (33)
Sexual	10 (28)	Yearly	4 (11)	<1 year	1 (3)

### Procedures

Data were collected using in-depth open interviews, in order to support the explorative character of this study (Bryman 2008). Professional interviewers were selected and trained for the study. The training consisted of an introduction to interparental violence, the influence of interparental violence on children, a discussion about the topic lists, interviewing victims of interparental violence, and interviewing children. Furthermore, arrangements were made with the interviewers concerning the safety of the situation during the interview. The interviewers were informed about potential risks associated with interviewing the participants.

The interviewers contacted the participants by telephone to make an appointment for the interview. Informed consent was granted during the recruitment of mothers and again at the beginning of each interview, as the interviewers explained the goals of this study. In addition, mothers were asked to explain the goals of the interview to their children. At the beginning of the interview, the interviewers explicitly told the participants that their information was to be used confidentially and anonymously. Additional measures were taken with regard to follow-up care for the participants. The interviewers offered to call several days after the interview to inquire about how the interviewees felt and how the interview had affected them. Several mothers appeared to perceive little or no support. The interviewers provided them with information about the domestic-violence support center.

The duration of the mother interviews ranged between 60 and 180 min (*M* = 101.97, *SD* = 29.60). For children, the duration ranged between 16 and 125 min (*M* = 61.18, *SD* = 23.04). Of all interviews, 58 % were conducted in the home of the participant, and 42 % were conducted in other settings (e.g., a women’s shelter or community center). These settings were selected according to the preferences of the participants. The interviewers evaluated all of the settings positively for the mothers, and they evaluated 98 % of the settings positively for the children. Before the interview, the interviewers invested time in creating a safe and trustworthy atmosphere. The interviewers stayed for a while after the interview, in order to end the session positively. The average duration of the visit was therefore 140 min (*SD* = 56.15) for mothers and 109 min (*SD* = 86.10) for children.

The interviews with the mothers were conducted in Dutch in 69 % of the cases; the mother’s native language was used in 22 % of the interviews, and 8 % of the interviews were conducted in both the native language and Dutch. Dutch was used in 89 % of the interviews with the children. In one interview, both the native language and Dutch were used. All interviews conducted in other languages were translated into Dutch by the interviewers. With permission from the participants, 94 % of the mother interviews and 91 % of the child interviews were recorded. Other participants gave interviewers permission to take notes. The recorded interviews were converted verbatim in transcripts by the interviewers. The six unrecorded interviews were converted in transcripts, based on extensive notes.

### Measures

Two topic lists were developed, one for the mother interviews and one for the child interviews. The selection of topics was based on a review of the literature (Lünnemann et al. 2011), as well as on interviews with social workers experienced in the field. In addition, topic lists from other studies that focused on immigrant families were considered (Dijkstra 2000; Lamers-Winkelman et al. 2007; Pels 2000; Skinner et al.^2005^). The concepts of the topic lists were controlled by an expert and a board of experts. The second version was evaluated with a team of experienced interviewers with respect to its content and structure. The lists were subsequently adjusted based on test interviews with mothers and children from various ethnic groups. In general, both interviews and respondents evaluated the test interviews positively. Slight adjustments were made with regard to the structure of the interviews and clarification of example questions.

The topics addressed in the mother interviews included the history of violence, parenting during and after interparental violence, and perceived support. Based on the narratives of the mothers the experienced violence was classified into two categories: (1) mild to moderate and (2) severe to very severe. Mild to moderate violence refers to sporadic physical violence causing none to moderate injuries and moderate psychological violence, such as humiliation and control. The severe to very severe category refers to monthly, weekly or daily physical violence that often resulted in severe injuries and severe psychological violence, like isolation, severe humiliations, and complete control.

The topics of the child interviews included the child’s exposure to violence, the impact of violence on the child, the child’s relationship with parents, future perspective, and perceived support. The language and content of the child interviews were adapted in interviews with the youngest children.

### Data Analyses

We used a combination of top-down and bottom-up coding in order to achieve saturation (Bryman 2008), thus establishing a hierarchical coding system that would reflect the goals of this study. Based on previous studies and the topic lists, we established a hierarchical central coding system, which we extended or adapted as necessary, based on induction from the interview materials.

We used the software program (ATLAS.ti 1999) to code the fragments and analyze data. The coding was performed by one member of the research team. To improve reliability, several members of the research team held discussions about the application of codes, thereby ensuring proper coding and the saturation of the codebook. This was an intensive period, in which the team discussed the consistency of the application of codes for specific fragments of the interviews. In addition, other researchers in the team systematically monitored the coding in order to determine whether interview fragments had been coded consistently and consequently across several interviews.

To assess the differences between the narratives of mothers and those of their children, we printed and compared the coded fragments. For the analysis, we subdivided the fragments into those related to the period during the interparental violence and those related to the period thereafter. The primary codes for the child’s exposure to interparental violence are children’s exposure to the interparental violence or, more specifically, the violence used by paternal caregivers and mothers. For child functioning, the primary codes are as follows: child abuse, children’s coping behaviors, and children’s problems (emotional and behavioral problems). The primary codes for parent functioning are the atmosphere in the home and parenting dimensions of support and control. In addition, for all discrepant findings exploratory analyses were performed to investigate relations with the severity of the violence and children’s age at the time of the interview. Patterns in discrepancies for type of violence and age of the children are only discussed in the results section when apparent; see Table 2 for an overview of all analyses. When multiple children within the same age group participated, we first compared their narratives. No differences were observed and their (aggregated) narratives were compared to the narratives of their mothers. In two families the two participating children did not belong to the same age group. In these cases analyses were conducted separately for the mother and the younger child and the mother and the older child, resulting in slightly different numbers (*n* = 38 instead of *n* = 36; mild to moderate (*n* = 18), severe to very severe (*n* = 20)) and children’s age at time of the interview [<16 (*n* = 23), ≥16 (*n* = 15)].

**Table 2:** Overview of the discrepancies between narratives of mother-child pairs considering the type of violence [Mild to moderate (*n* = 18), Severe to very severe (*n* = 20)] and age of the child [<16 (*n* = 23), ≥ 16 (*n* = 15)] during the period of interparental violence

Discrepancies in	Type of violence		Age child	
	Mild to moderate	Severe to very severe	<16 (*n*)	≥16 (*n*)
Exposure				
Child’s exposure	33 % (6)	45 % (9)	39 % (9)	40 % (6)
Role mother	50 % (9)	20 % (4)	44 % (10)	20 % (3)
Explanatory factors				
Problems father	71 % (12)	85 % (17)	73 % (17)	80 % (12)
Child functioning				
Abuse: psychological	50 % (9)	25 % (5)	48 % (11)	20 % (3)
Abuse: physical	17 % (3)	50 % (10)	39 % (9)	27 % (4)
Coping behavior: avoidance	28 % (5)	30 % (6)	22 % (5)	40 % (6)
Problems: internalizing problems	61 % (11)	40 % (8)	44 % (10)	60 % (9)
Problems: externalizing problems	22 % (4)	5 % (1)	13 % (3)	13 % (2)
Parental functioning				
Support and control	22 % (4)	35 % (7)	30 % (7)	27 % (4)

In the results, pronouns are used to determine quantity (Sandelowski 2001). *Few* and *rarely* indicate that something occurred in less than 20 % (*n* ≤ 7) of the participants; *several* and *minority* refer to events recurring in between 20 and 49 % (7 < *n* < 17) of the interviews, *often* and *majority* between 50 and 80 % (18 < *n* < 29), and *many* and *frequently* refer to more than 80 % (*n* ≥ 29) of the interviews. In the results, paternal caregivers are described as paternal caregivers or fathers. We use quotations from mothers and children in order to illustrate the results, noting additional information about the respondent (e.g., mother or child, gender of child, age, and ethnic background).

Although the sample consisted of respondents of different ethnic background, we did not include ethnicity as a factor in the analyses because of the small size of the subgroups. However, the broader study of which our subsample was drawn, pointed out that discrepancies between the narratives of ethnic groups with regard to parent and child functioning were negligible (Pels et al. 2010).

## Results

### Narratives Referring to the Period of Interparental Violence

#### Interparental Violence and the Exposure of Children

We assessed the child’s exposure and the role of the mother regarding the interparental violence. In general, both mothers and children frequently reported that the children had been exposed to interparental violence. Nevertheless, we identified differences between the narratives of mothers and those of their children regarding the child’s exposure to interparental violence for the majority of mother-child pairs. In several cases, the children’s reports were more specific than those of their mothers were. Their mothers tended to underreport the situations in which their children had been witnesses. The children had apparently witnessed more of the violence than their mothers had assumed.

Mother: *The children never saw it [the violence].* (27, Dutch)

Child: *It happened in the kitchen, it started with calling names, and then he hit her.* (Girl, 14, Dutch)

Furthermore, we identified differences between the narratives of mothers and those of their children with regard to the violence used by the mothers. Several children referred to mutual violence, while their mothers emphasized only the violence of their partners. This discrepancy seemed mostly present in families that experienced mild to moderate types of violence and in children below the age of 16 (Table 2).

Mother: *It [the violence] was really focused on me.* (32, Hindustani)

Child: *My father hits, but my mother is far worse. When she starts screaming, everybody hears her. (…) When my father asked for money, I heard my mother yelling and calling my father names. Then she kicked my father out of the house. My mother hit my father, and my father hit my mother, but he could not handle her. He is too scared.* (Boy, 11, Hindustani)

#### Explanatory Factors for Interparental Violence

We assessed several factors that may have resulted in the initiation or amplification of the interparental violence. First, many of the mothers reported that problems of their partners might have fuelled their violent behaviors. The majority of the children did not refer to their paternal caregivers’ problems, but several of them did: “*I think that the domestic violence was largely due to my father’s alcoholism, because every time he was aggressive he had been drinking*” (girl, 17, other ethnic background). Within the mild to moderate group a few children were more aware of their fathers’ problems than their mothers had reported (Table 2).

Mother: *We did not find it necessary to tell the truth about my partner’s gambling addiction.*

Interviewer*: Did you have a specific reason for not telling your children the truth?* Mother: *Yes, I think that the children need not to know about their father’s mistakes (…). He is very important to us.* (33, Turkish)

Child: *I do not think it is normal for a man with money to go to a coffee shop. That is already a problem.* Interviewer: *What do you think what a man does with that money?* Child: *I think gambling.* (Boy, 13, Turkish)

Second, many mothers reported that they perceived no support from relatives. Relatives ignored, supported, or even maintained the partner’s violence–*“If he was going to hit me in the bedroom, his mother came inside and closed the door so I could not escape, she just pulled at the door*” (mother, 41, Moroccan). The majority of the children did not mention such negative roles on the part of their relatives. However, few children were aware of the negative contributions of their relatives to the context of interparental violence– “*They had been married off* via *family. My father did not want to marry my mother and my mother thought she had no choice*” (Girl, 22, Hindustani).

#### Child Abuse and Child Functioning During the Period of Interparental Violence

We assessed several aspects of child functioning during the period of interparental violence: child abuse, children’s coping behaviors, children’s feelings, and children’s problems. Many of the mothers and children reported occurrences of child abuse. We identified several discrepant reports with regard to psychological child abuse. Several mothers seemed to underestimate the level of verbal or physical abuse of children by their paternal caregiver compared to their children. Additionally, several children did not frame their fathers’ behavior in these terms, although their mothers did report psychological abuse (e.g., child neglect on the part of the father).

Mother: *He [father] gave them nothing, absolutely nothing.* (56, Surinamese-Antillean)

Interviewer: *Were you a victim of or involved in the violence?* Child: *Mm, no.* (Girl, 21, Surinamese-Antillean)

This discrepancy seemed more often apparent in families exposed to mild to moderate violence, compared to severe to very severe violence, and in children below the age of 16, compared to older children (Table 2). Discrepancies regarding physical abuse, however, appeared more in families that were exposed to severe to very severe types of violence (Table 2): several mothers reported lower levels of physical abuse of children by their paternal caregivers compared to their children.

Mother: *For a while, he was not allowed to hit me. Therefore, he tried to hit my daughter. However, I said: ‘if you touch her, I will kick you dead totally.’* Interviewer: *So he never hit her?* Mother: *He never had the chance to hit her, because I told him. I said, ‘If you touch my children, you touch me’. So he did not dare.* (35, Moroccan)

Child: *He, eh …always hit me. When I was around six, there was a spider in our shower. And, eh … I woke him and asked him “would you please get it out of the shower,’ but he did not want to. I started crying and, eh… then he became angry and he just pulled my nose very hard, so it started to bleed.* (Girl, 12, Moroccan)

The minority of the mother-child pairs had different narratives with regard to the child’s coping behaviors. In general, children commonly reported that they had avoided situations of parental violence, mostly by fleeing to their room, sometimes by intervening or seeking external help. Several mothers reported less avoidance of the violence than their children did. These mothers were primarily focused on the externalizing coping behaviors of their children (e.g., protecting their mothers by attacking their paternal caregivers). In contrast, their children emphasized the avoidance of the violence by going to their rooms or staying at school.

Interviewer: *So the children did see that he was banging on the door?*

Mother: *Yes, when they were bigger, (…) the children opened the door and said: ‘Dad, get out!*’ (56, Surinamese-Antillean)

Interviewer: *When there were problems, what did you do?* Child*: Oh, then I left.* (Girl, 21, Surinamese-Antillean)

With respect to the impact of interparental violence on the children’s feelings, both mothers and children frequently reported that children had been sad, frightened, or angry. We found no differences between the narratives of mothers and those of their children with regard to the children’s feelings.

Nearly half of the mother-child pairs showed discrepancies between narratives with regard to the children’s problems. Several children reported having experienced more internalizing problems than their mothers had reported. First, several mothers underestimated or did not mention the internalizing problems of their children. Second, a few mothers, especially of those exposed to mild to moderate types of violence (see Table 2), reported more externalizing problems than their children.

#### Parental Functioning During the Period of Interparental Violence

We examined two aspects of parent functioning during the period of interparental violence: the atmosphere in the home and parental support and control. First, we found no clear discrepancies between the narratives of mothers and children regarding the atmosphere in the home. All of the mothers and children reported that they had perceived an atmosphere with high levels of tension, fear, or agitation during the period of interparental violence.

With respect to parenting, mothers frequently reported that they had lacked the time or energy for properly supporting and/or controlling their children Several of the children did not mention issues regarding parenting. In the group that did, we identified several discrepancies with the mothers’ narratives. A few children were more negative in their reports than their mothers were. These children, mainly exposed to severe to very severe violence (Table 2), reported higher levels of abreaction or inconsistency between parents.

Mother: *What was not allowed was not allowed. It was not that the children were allowed to do more when they asked me or their father. That has always been a straight line.* (38, Dutch)

Interviewer: *What kind of punishment did you receive?* Child*: That I had to stay in my room when I was not in school. (…)* Interviewer*: And what did your mother think of that?* Child: *She did not like it, when my father was gone she called me down.* (Boy, 14, Dutch)

In contrast, several children seemed more positive than their mothers were with regard to parenting. These mothers reported primarily about their difficulties with parenting, while their children emphasized the positive attention they had received.

Mother: *Parenting did not go well. (…) I did not even know what parenting was, because I was struggling with my own problems. (…) I really did not give my daughter attention in terms of parenting or education.* (42, Turkish)

Child: *They didn’t fail in our upbringing; they did give everything to us.* (girl, 19, Turkish)

### Narratives Referring to the Period After the Interparental Violence

The majority of the mothers were not living with a partner at the time of the interview. They had been living alone for between 2 to 144 months (*M* = 47.35, *SD* = 43.92). Several mothers were living with either a new or the same partner. Although the mothers were no longer experiencing interparental violence, the majority reported they still had problems with their former partners. For example, some partners harassed the mothers by continuously contacting them or visiting their home. Other partners failed to keep to agreements made with regard to the care of the children.

#### Child Functioning After the Period of Interparental Violence

We investigated the ways in which child functioning (i.e., coping behavior, feelings, and problems) had been affected after the period of interparental violence. Often mothers did not mention their children’s coping behavior after the period of interparental violence. The few mother-child pairs that both reported on the child’s coping differed in that the mothers reported their children to have moved on, while their children told they were still working on dealing with their past.

Several mothers did not report anything about their children’s feelings. We found several discrepancies between the other mother-child pairs (see Table 3). The mothers concerned were often exposed to severe to very severe violence. First, a few mothers did not recognize their children’s anger towards their fathers. Second, a few mothers reported about their children’s anger towards or fear of their paternal caregivers or mothers, while their children, especially those above the age of 16, expressed the fear that the same might happen to their mothers again or to themselves later in life.

**Table 3:** Overview of the discrepancies between narratives of mother-child pairs considering the type of violence [Mild to moderate (*n* = 18), Severe to very severe (*n* = 20)] and age of the child [<16 (*n* = 23), ≥ 16 (*n* = 15)] after the period of interparental violence

Discrepancies in	Type of violence		Age child	
	Mild to moderate (*n*)	Severe to very severe (*n*)	<16	≥16
Child functioning				
Coping behavior: disclosure child	11 % (2)	10 % (2)	9 % (2)	13 % (2)
Feelings: sadness, anger, fear	6 % (1)	15 % (3)	13 % (3)	13 % (2)
Feelings: afraid for the future	6 % (1)	15 % (3)	4 % (1)	20 % (3)
Feelings: distrust	33 % (6)	30 % (6)	22 % (5)	47 % (7)
Problems: parentification	17 % (3)	10 % (2)	13 % (3)	13 % (2)
Parental functioning				
Atmosphere	22 % (4)	10 % (2)	22 % (5)	7 % (1)
Support and control	30 % (5)	45 % (9)	43 % (10)	26 % (4)

Mother: *My daughter felt that I had to stand up for myself. (…) She will never accept a man treating her like that.* (49, Hindustani)

Child: *You do become afraid of it, because you start to think, but it depends on your man.* Interviewer: *So you are afraid that the same will happen to you?* Child: *Yes.* (Girl, 16, Hindustani)

Differences between the narratives of mothers and those of their children were common with respect to problems related to the past period of interparental violence. First, several mothers did not recognize their children’s distrust toward people in general or men in particular. This difference seemed more often present in cases where children were above the age of 16 (Table 3). Second, several children reported higher levels of parentification than their mothers had reported.

#### Parental Functioning After the Period of Interparental Violence

With respect to parental functioning after the period of interparental violence we examined the atmosphere in the home, support and control. Although several mothers did not report about the atmosphere after the period of interparental violence, other mothers and their children often reported a more relaxed or quiet atmosphere in the home– “*When he was gone, I experienced a sort of light in my house and life*” (mother, 37, Surinamese-Antillean). Mother-child pairs rarely differed in their accounts of the home atmosphere. A few children, mainly exposed to mild to moderate types of violence and below the age of 16, emphasized an increased level of mother-child intimacy, while their mothers only mentioned increased quietness.

Mother: *It is calmer now.* (40, Turkish)

Child: *We have more time for each other and do more things together; in the past, we did not want to do these things.* (girl, 18, Turkish)

Several of the mother-child pairs reported differently regarding parental support and control. Several children evaluated their mothers’ parenting behaviors less positively than their mothers did. These mothers told to have become stronger as parents, while their children highlighted their mothers’ lack of attention towards them. In contrast, a few children, often exposed to more severe violence and below the age of 16 (Table 3), reported more positively about parenting than their mothers did.

## Discussion

The purpose of the current study is to investigate similarities and differences between the narratives of mothers and their children with regard to the children’s exposure to interparental violence and its impact on child and parental functioning. This study is necessary in order to enhance knowledge about these discrepancies and, eventually, to provide sufficient support to families exposed to interparental violence. Previous studies have rarely examined such discrepancies. Based on the Intergenerational Stake Hypothesis (Bengtson and Kuypers 1971; Giarrusso et al. 1995), the Social Structural Model (Risman and Park 1988; Shapiro 2004), and the Attribution Bias Context Model (De Los Reyes 2011; De Los Reyes and Kazdin 2005), we expected that mothers and children would differ in their narratives, due to discrepant motivations, perceived expectations, and informant attributions. In summary, the results revealed discrepancies between the narratives of mothers and those of their children with regard to the children’s exposure to interparental violence and its impact on child functioning. Narratives about parental functioning were more similar. Furthermore, exploratory analysis indicated that discrepancies often were related to the severity of the violence and, to a lesser extent, to the age of the children.

We found discrepancies between the narratives of mothers and children with regard to the child’s exposure to interparental violence. Children reported more violence than their mothers had assumed, as well as more violence of mothers towards fathers than their mothers did. These findings are consistent with previous studies, which show that parents tend to underestimate or fail to notice their children’s exposure to interparental violence (e.g., Margolin and Gordis 2004). For example, parents might not be aware of the presence of their children, because they were hiding or pretending to be asleep (Holden 2003). Nevertheless, based on the Intergenerational Stake Hypothesis and the Social Structural Model, mother-child disagreement concerning the child’s exposure to interparental violence could be explained by inter-generational discrepancies in terms of motivation and expectations. For example, parents hope that their children are not exposed to interparental violence. They want to protect their children (Peled and Gil 2011). Despite this, the powerlessness that they experience, combined with the perceived expectations of society with regard to parenting, might lead mothers to disregard their children’s exposure to the violence, as well as their own contribution to the violence. In other words, the underreporting of children’s exposure to violence may be partly due to social expectations (Risman and Park 1988; Shapiro 2004). The higher level of mutual violence in child reports compared to mother reports could also be explained by the Attribution Bias Context Model (De Los Reyes and Kazdin 2005; De Los Reyes 2011). Children might report more mutual violence than their mothers do because they attribute the causes of interparental violence to their mother’s disposition. Remarkably, this discrepancy appeared to occur more often when the fathers’ violence was less severe and when children were younger. An explanation might be that father violence overshadows the mothers’ violence more when it is more severe. Additionally, assuming that they have a longer history of experiencing violence between their parents, older children might tend to relativize their mothers’ share in the violence more compared to younger children.

With regard to child functioning, mothers and children reported differently about the impact of interparental violence. Children reported more physical and less psychological child abuse than their mothers reported. This is consistent with a previous study that also found high levels of disagreement between the reports of mothers and those of their children with regard to child abuse. Mothers reported less severe physical violence, more psychological aggression, and more neglect than their children reported (Chan 2012). Discrepancies between the narratives of mothers and children with regard to physical or psychological violence may also be associated with the mother’s motives and perceived societal expectations about good parenting behaviors. Moreover, higher levels of discrepancies in families exposed to severe to very severe types of violence, compared to families exposed to mild to moderate types of violence, could be explained by higher levels of family stress and conflict within these families (Grills and Ollendick 2003). Differences in narratives about psychological violence could be due to discrepancies in the understanding of parental behavior between mothers and children. Young children may less easily recognize psychological violence as violence, which may explain why the mother-child narratives about psychological violence were more discrepant with younger than with older children. Psychological child abuse might also be more subtle in families exposed to mild to moderate violence. This could explain the finding that the mother-child discrepancy was more apparent in these families. In addition, children reported more avoidant coping behaviors, more internalizing and fewer externalizing problems than were reported in the narratives of their mothers. This is generally consistent with previous studies on children’s behavior problems. Internalizing problems are difficult to recognize for parents, as they are often not clearly visible (e.g., Sourander et al. 1999). This might also explain the discrepancies between narratives concerning avoidant coping behaviors. Moreover, the discrepant narratives with regard to the externalizing problems of the children could be explained according to the Attribution Bias Context Model. It could be that children tend to attribute the causes of their problems to the context, while mothers tend to highlight the dispositions of their children. Why this discrepancy occurs less in situations of more severe violence might be explained by the fact that mothers expect more aggression-intergenerational transfer of the violence-in such a context (e.g., Pels et al. 2010).

In contrast to child exposure and child functioning, we identified fewer differences between the narratives of mothers and children with regard to the impact of interparental violence on family functioning. The higher levels of agreement between mothers and children could be explained by the lesser risk of attribution bias due to the fact that both parents and children function as actors in situations of family functioning.

Finally, it is important to emphasize that discrepancies between the narratives of mothers and children seemed more prevalent with regard to the period of interparental violence than with regard to the period thereafter. The level of stress during the period of interparental violence could explain this difference. A stressful atmosphere might have limited the mother’s sensitivity to other family members, thus increasing the discrepancies between the narratives of family members (Shek 1998). Similarly, discrepancies between the reports of parents and children are associated with high levels of family stress and conflict (Grills and Ollendick 2003). For most of the families in the current study, family stress and conflict had been more apparent during the period of interparental violence than afterwards.

This study adds to the still limited evidence base regarding different perceptions of children and parents regarding their experiences with interparental violence and the influence of the violence on child and parent functioning. Understanding these discrepant perspectives is extremely important for the prevention, assessment and treatment of interparental violence and its consequences for children’s development. When professionals and non-professionals are more aware of the discrepant narratives, a risk assessment might be made sooner and better to ensure the safety of the child and to provide sufficient support. The use of in-depth open interviews about interparental violence helped to provide rich information about family members’ experiences of interparental violence and the discrepancies between mothers and their children exposed to such violence.

Despite these advantages, it should be noted that the participants varied with respect to the amount of time that had passed since the interparental violence occurred. Narratives about interparental violence that have taken place several months earlier could be expected to differ from those about events of several years back. Furthermore, although we examined patterns for severity of violence and age of the children at the time of the interview, it must be mentioned that these exploratory analyses were based on very small sample sizes. In addition, the accents in the mother and child interviews sometimes differed (more on violence in the mother interviews and on the influence of the violence on child functioning in the child interviews). This was largely due to the open interview approach and the tailoring of the interviews to the stories of the respondents. Nevertheless, this could have led to either under-reporting or over-reporting of discrepancies between the narratives of mothers and children. Furthermore, this limited the possibilities to explore the underlying dynamics explaining the discrepancies.

Our study has provided insight into important aspects of similarities and discrepancies in narratives regarding parent and child functioning in the context of family violence. Ideally, future studies should include quantitative research designs and representative samples of the population, in order to improve the quantitative reliability and generalizability of the findings. This could prove difficult, however, especially due to the problems associated with recruiting respondents who have experienced interparental violence and, the sensitivity of the topic. Nevertheless, investing in such an undertaking is important, as it could help to create a better foundation for improving social and assistance services for the families involved.

The increased knowledge about discrepancies between the narratives of mothers and those of their children with regard to exposure to interparental violence enhances understanding of the complex family dynamics and possible influence of children’s age and severity of violence. This has implications for the development of effective strategies for supporting mothers and children who have been victim of interparental violence. Our findings suggest the necessity of including multiple informants in clinical screening and intervention programs in order to obtain complete information about the impact of interparental violence. In the Netherlands, an example of good practice is a parallel treatment for parents and children (Berger et al. 2004). Furthermore, both mothers and children reported that mothers had struggled with parenting during and after the period of interparental violence. It is therefore important to offer them parenting support, also to raise their awareness of the consequences of interparental violence on the functioning of their children (Pels et al. 2010).

In summary, the current study highlights the similarities and discrepancies between the narratives of mothers and children with regard to the exposure to and the impact of interparental violence. The study indicates that severity of interparental violence and age of the children might influence these discrepant narratives. Future studies and clinical settings should devote more attention to these issues, in order to improve understanding with regard to the complex family dynamics in the context of family violence and, ultimately, to provide sufficient support.
